# Cassava consumption and the occurrence of cyanide in cassava in Vietnam, Indonesia and Philippines

**DOI:** 10.1017/S136898001900524X

**Published:** 2020-05-22

**Authors:** Jatziri Mota-Gutierrez, Gerard Michael O’Brien

**Affiliations:** 1 Department of Agricultural, Forest and Food Sciences, University of Turin, 10095 Grugliasco, Italy; 2 School of Biomedical, Nutritional and Sports Sciences, Newcastle University, Newcastle upon Tyne, UK

**Keywords:** Cassava consumption, Cyanogenic glucosides, Cyanide, South-east Asia, Public health risk

## Abstract

**Objective:**

To make a tentative assessment of the consumption of cassava in three countries in South-east Asia and the cyanogenic potential (CNp) of the crop as a possible food safety issue.

**Design:**

We used data from the Ministry of Health in Vietnam and Statistics Authorities in Indonesia and Philippines (mean household consumption per province) to assess cassava consumption. Conversions of units were needed to facilitate the comparison of cassava consumption between countries. The most up-to-date data available regarding both cassava consumption and the CNp of cassava grown in the respective countries were assessed.

**Settings:**

Vietnam, Indonesia and Philippines.

**Participants:**

Respondents from provinces in Vietnam (nineteen), Indonesia (thirty-three) and Philippines (eighty-one) were asked to complete a recall questionnaire detailing either the previous 24-h’ or the 7-d’ cassava consumption.

**Results:**

Among the three countries, available data indicated that the highest median cassava-consumption figures *per* *capita* were from Indonesia and the Philippines (9·01 and 7·28 g/capita per d, respectively), with Vietnam having the least (1·14 g/capita per d). Published information regarding the CNp of cassava in the three countries was limited.

**Conclusions:**

While the findings of the present study are somewhat limited by a lack of available information regarding both the extent of cassava consumption and the CNp of cassava consumed in the three countries, it appears likely that cyanogen intake arising from cassava consumption among the three countries exceeds the FAO/WHO Provisional Maximum Tolerable Daily Intake, although any risk to public health appears limited to a minority of provinces in each country.

Food security is considered a major concern for Asia. In 2017, it was calculated that 821 million people in the world – some 11 % of the total global population – were undernourished. While the continent most affected by undernutrition was Africa, with a prevalence of undernourishment of nearly 21 %, the estimated prevalence of undernourishment in South-eastern Asia in 2017 was 9·8 %, which was virtually unchanged from 2015 to 2016^(1)^. While this is somewhat below the reported overall prevalence of undernourishment for Asia (11·4 %), it is nonetheless a matter of concern. As a result, several strategies have been used to reduce the rate of undernourishment, such as providing nutritional supplements, nutritional information campaigns, biofortification, access to health care, and research and development to increase crop yields^(2,3)^. According to Warr^(4)^, the access dimension of food security is an important determinant of undernourishment within developing countries, especially in Asia. Cassava (*Manihot esculenta* Crantz) is considered one of the most efficient producers of carbohydrates under poor agricultural conditions, an advantage that is believed to have led to its adoption in some countries as a source of dietary energy as a ‘food security food’^(5)^. According to the US National Nutrient Database for Standard Reference, the macronutrient value of the edible portion of fresh cassava root (root parenchyma) per 100 g is 38·6 g carbohydrate, 1·36 g proteins and 0·28 g fat^(6)^. Therefore, as a rich source of dietary carbohydrate, cassava may play an important role in preventing hunger^(7)^.

Since the introduction of cassava in South-east Asia, this root crop has been intensively grown in various parts of this subregion of Asia. According to recently published figures, in 2017, the major cassava-producing country in Asia, in terms of overall amount of roots per country, was Thailand (30·9 million tonnes), followed by Indonesia (19·0 million tonnes), Cambodia (10·5 million tonnes), Vietnam (10·3 million tonnes), mainland China (4·8 million tonnes), India (4·1 million tonnes), Philippines (2·8 million tonnes), Lao People’s Democratic Republic (2·2 million tonnes) and Myanmar (0·3 million tonnes)^(8)^. In terms of utilisation of cassava as human food, the most recent data available indicate that, in 2013, an estimated 11·73 million tonnes were utilised in this way in Indonesia, with smaller quantities utilised in other South Asian countries, such as India (6·93 million tonnes), mainland China (2·64 million tonnes), Philippines (2·29 million tonnes), Thailand (0·87 million tonnes), Vietnam (0·74 million tonnes) and Myanmar (0·57 million tonnes). In addition, Indonesia was reportedly the country whose inhabitants sourced the highest energetic availability from cassava and its products (552·288 kJ/capita per d), followed by the Lao People’s Democratic Republic, Cambodia, the Philippines, Thailand, Myanmar and Vietnam (330·536, 292·88, 267·776, 167·36, 125·52 and 92·048 kJ/capita per d respectively)^(8)^. Interestingly, cassava consumption from 1961 to 2013 has increased in Philippines and Vietnam, while a decrease is observed in Indonesia, as shown in Fig. 1^(8)^.

**Fig. 1:**
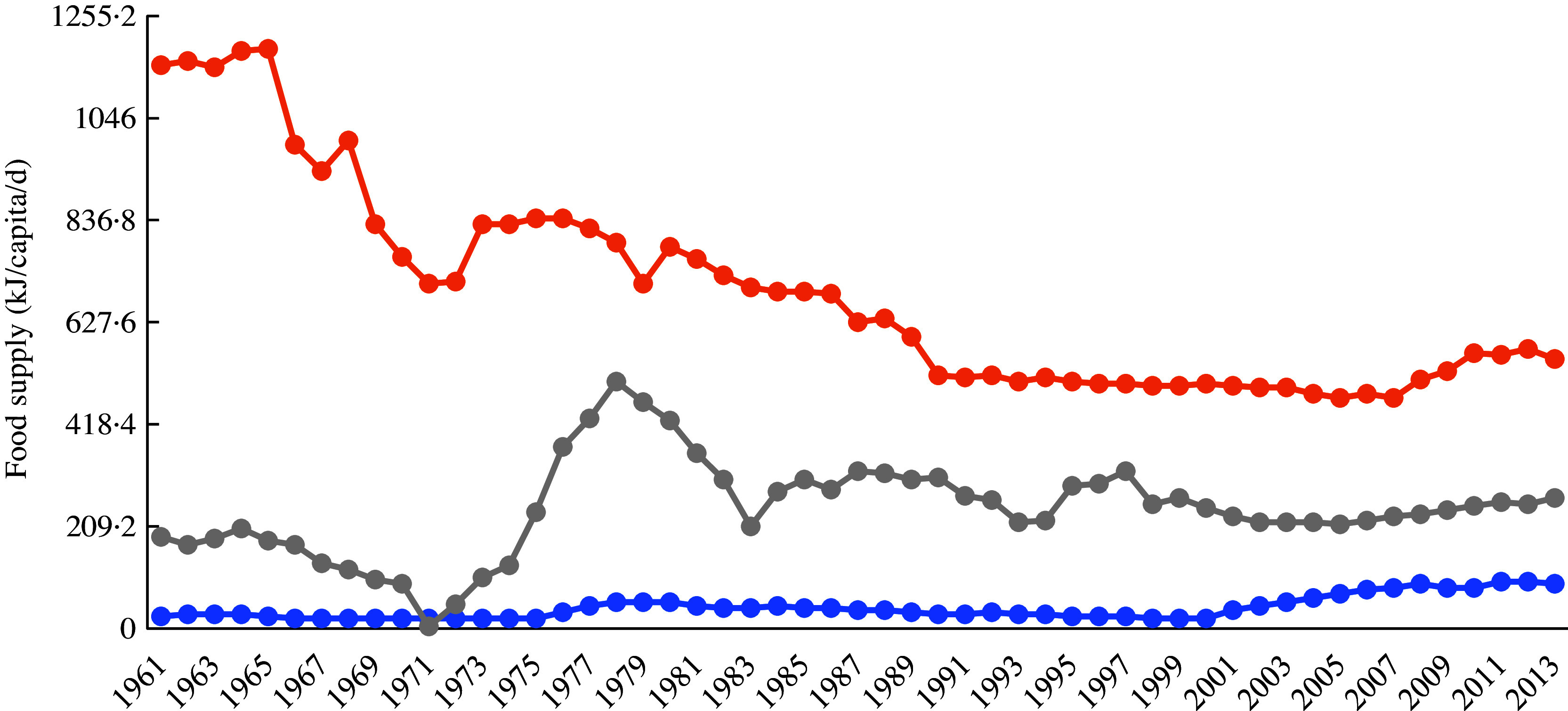
Cassava supply trends (kJ/capita per d) at national/regional level from 1961 to 2013. 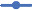, Vietnam; 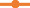, Indonesia; 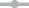, Philippines

Importantly, cassava roots naturally contain potentially toxic cyanogenic glucosides, mainly linamarin, which break down and produce hydrogen cyanide (HCN), apparently as a defence mechanism for the crop plant against predators^(9)^. Both plants and animals possess some ability to detoxify cyanide^(10)^. However, the concentration of cyanogens, or cyanogenic potential (CNp) as it is known, and thus the potential amount of HCN that can be released, is variable, and if roots of high-CNp (so-called ‘bitter’) cultivars are not detoxified adequately during processing into cassava food products, they may pose a serious toxicological threat^(11,12)^. Depending on their handling and processing history, cassava products may contain varying amounts of cyanogenic glucosides, cyanohydrin (an ‘intermediate’ form of cyanogen) and/or HCN. Both cyanogenic glucosides and cyanohydrin can yield HCN. Therefore, when assaying the cyanogen content of cassava, it is a common practice to quantify all three types and express them all together either as total hydrocyanic acid (total HCN) or as CNp, in ‘mg HCN equivalents per kg^’(13)^.

In terms of the potential health-related effects of cyanogens in cassava, the scientific literature indicates that symptoms of acute intoxication may arise from the consumption of unprocessed high-CNp cassava roots^(14)^. In addition, cassava-consuming groups whose protein intake is considered inadequate may become vulnerable to particular problems. Banea-Mayambu *et al.*^(15)^ have linked exposure to insufficiently processed high-CNp cassava with growth retardation in children in the Democratic Republic of Congo, on account of amino-acid depletion through the body’s conversion of cyanide into thiocyanate (a necessary part of the cyanide-expulsion process) drawing on S taken from the body’s pool of S-containing amino acids methionine and cysteine. In 2011, the Joint FAO/WHO Expert Committee on Food Additives (JECFA) established health-based guidance values for dietary cyanide, including a Provisional Maximum Tolerable Daily Intake (PMTDI) of 20 μg/kg body weight, to allow calculation of health risks^(16)^.

With the aim of ensuring consumer safety, internationally recognised bodies such as FAO have declared maximum CNp levels for fresh and processed cassava^(17)^, although there appears to be some disagreement between different bodies as to what constitutes a ‘safe’ CNp^(18)^. This review aimed to assemble and discuss the latest available data regarding cassava consumption and to highlight the reported CNp levels of cassava used for human consumption as a potential food safety issue in Vietnam, Indonesia and Philippines.

## Methods

### Literature search

Published reports were searched using the terms ‘«cassava intake»’, ‘«cassava consumption»’ AND ‘«Vietnam»’, OR ‘«Indonesia»’, OR ‘«Philippines»’, ‘«cyanide»’, ‘«cyanogenic potential»’, ‘«hydrocyanic acid»’, ‘«hydrogen cyanide»’, ‘«cyanogenic glucosides»’ AND ‘«cassava roots»’, ‘«sweet cassava»’, ‘«bitter cassava»’, ‘«fresh cassava»’ using electronic databases (Medline, EMBASE, PUBMED and Google Scholar). Relevant national databases were consulted for cassava-consumption data arising from food consumption surveys. While the focus was mainly on fresh cassava, information relating to consumption of dried cassava (cassava flour and *gaplek*) was also included. Additionally, published scientific literature was the source from which information regarding reported levels of cassava cyanogens from Vietnam, Indonesia and the Philippines was obtained.

### Methodology used to obtain reported cassava-consumption data

In each of the countries, the cassava-consumption data were obtained from household-level questionnaire surveys conducted by respective government ministries, these being the Ministry of Health in Vietnam^(19)^ and Statistics Authorities in Indonesia^(20)^ and the Philippines^(21)^. Respondents from different provinces in Vietnam (*n* 63 provinces), Indonesia (*n* 33 provinces) and Philippines (*n* 81 provinces) reported consumption of their households during either the previous 24 h or the previous 7 d. Table 1 shows an overview of the methodology used for each country. From these surveys, the mean *per capita* fresh cassava-consumption data for the three countries, by province, were obtained.

**Table 1:** Overview of the food consumption survey methods for Vietnam, Indonesia and Philippines

	Vietnam	Indonesia	Philippines
Institute	National Institute of Nutrition	The National Socioeconomic Survey	Bureau of Agricultural Statistics
Year of publication	2010	2014	2010
Study design	Cross-sectional	Cross-sectional	Cross-sectional
Data collection	2009–2010	September 2014	2008–2009
Subjects	Household and its members	Household and its members	Household and its members
Average household	4·3 people	ND	ND
Sample size	Sixty-three provinces	Thirty-three provinces	Eighty-three provinces
Sampling method	Population-based, Multistage cluster sampling: regions and provinces	Population-based, Census block: national and provinces	Population-based, Census block: national and provinces
Socio-economic/demographic	Yes	Yes	Yes
Dietary intake survey tool	24-h recall	7-d recall	7-d recall
Type(s) of cassava included	Fresh and dried	Fresh and dried	Fresh only
Duration	24-h recall	1-week period	1-week period
Units of measurement	g/capita per d	kcal/capita per d	kg/capita per year

ND, not determined.

Data reported by Vietnamese National Institute of Nutrition^(19)^, the National Socioeconomic Survey^(20)^ and Bureau of Agricultural Statistics^(21)^.

### Collection of cassava-consumption data

The data relating to fresh and dried cassava consumption of each country were entered in MS Excel spreadsheets. Arithmetic mean household *per capita* consumption of fresh cassava per province from Indonesia and Philippines was accessible via government ministry websites^(20,21)^. No access to raw data from these national bodies was provided; therefore, sd were not available. In the case of Vietnam, a complete root and tuber crop data set, expressed as arithmetic mean household *per capita* values per region and province, was provided by the National Institute of Nutrition^(19)^. The Vietnamese data set included only nineteen cassava-consuming provinces out of the sixty-three. Zero cassava consumption was recorded in the majority of provinces in Vietnam. It is worth noting that the cassava-consumption data from all three data sets were originally expressed in different measurement units. In Vietnam, they were expressed as g/capita per d; in Indonesia, they were expressed as kcal/capita per d; and in the Philippines, they were expressed as kg/capita per year. Hence, conversion of units to g/capita per d was required in order to facilitate comparison of cassava consumption between countries. Moreover, while dried cassava-consumption data from Vietnam and Indonesia were accessible expressed as mean consumption per province, no data regarding the consumption of dried cassava were provided by the Philippines Bureau of Agricultural Statistics. Inter-country comparison of fresh cassava consumption was performed by calculating the country median from the provincial means of the cassava-consuming provinces. No data regarding the consumption of other derived or secondary cassava products besides dried cassava such as chips, tapioca and noodles were reported in any of the three countries. Therefore, it was not possible to conduct inter-country comparisons of dried cassava or cassava products dietary consumption, owing to the lack of data from the Philippines.

### Recommended maximum cassava cyanogenic potential levels set by different authoritative bodies

In this review, we have compared reported CNp levels of fresh and dried cassava from Indonesia, and fresh cassava from the Philippines and Vietnam, with the maximum levels of CNp suggested by three different authoritative bodies. As regards dried cassava, in 1993 the WHO recommended a maximum CNp of 10 mg/kg as HCN for cassava flour^(22)^. Meanwhile, in Indonesia, cassava flour is permitted to contain up to 40 mg/kg as HCN^(23)^. As regards fresh cassava, the Codex Alimentarius Commission (CAC) defines ‘sweet’ (safe to consume after cooking) cassava as having a total CNp of below 50 mg/kg as HCN on a fresh weight basis^(24)^. Given that the typical range of parenchymal moisture content in cassava roots is approximately 56–75 %^(25)^ this would be equivalent to approximately 111–200 mg/kg on a dry weight basis, respectively. Needless to say, any variation in moisture content would also give rise to variation in the carbohydrate content of the roots.

### Statistical analysis

As indicated earlier, the ‘raw data’ relating to cassava consumption that were obtained in this study consisted of reported estimated regional/provincial mean values obtained from the relevant government ministry of each country. Prior to comparison of the data of the three countries, the data were tested for normality of distribution and equality of variance using Shapiro–Wilk and Levene’s tests, respectively. As non-parametric testing was the indicated outcome, the comparisons were carried out using Mood’s Test of Medians. Consequently, all fresh cassava dietary consumption values are given as median and interquartile range. Where a significant overall difference in dietary intake was indicated (*P* < 0·05) among the three countries, Mood’s Test was applied in pair-wise *post hoc* mode, using the Bonferroni’s adjustment. The functions and packages used to perform statistical analyses were acquired through the *median_test* function in the *coin*™ package and the function *pairwise Median Test* in the *rcompanion* package of R™ version 3.5.1.

## Results

Key features pertaining to data obtained from the three countries (including the number of provinces per country that featured in the study) are presented in Table 1.

### Reported cassava consumption in Vietnam

**Fig. 2:**
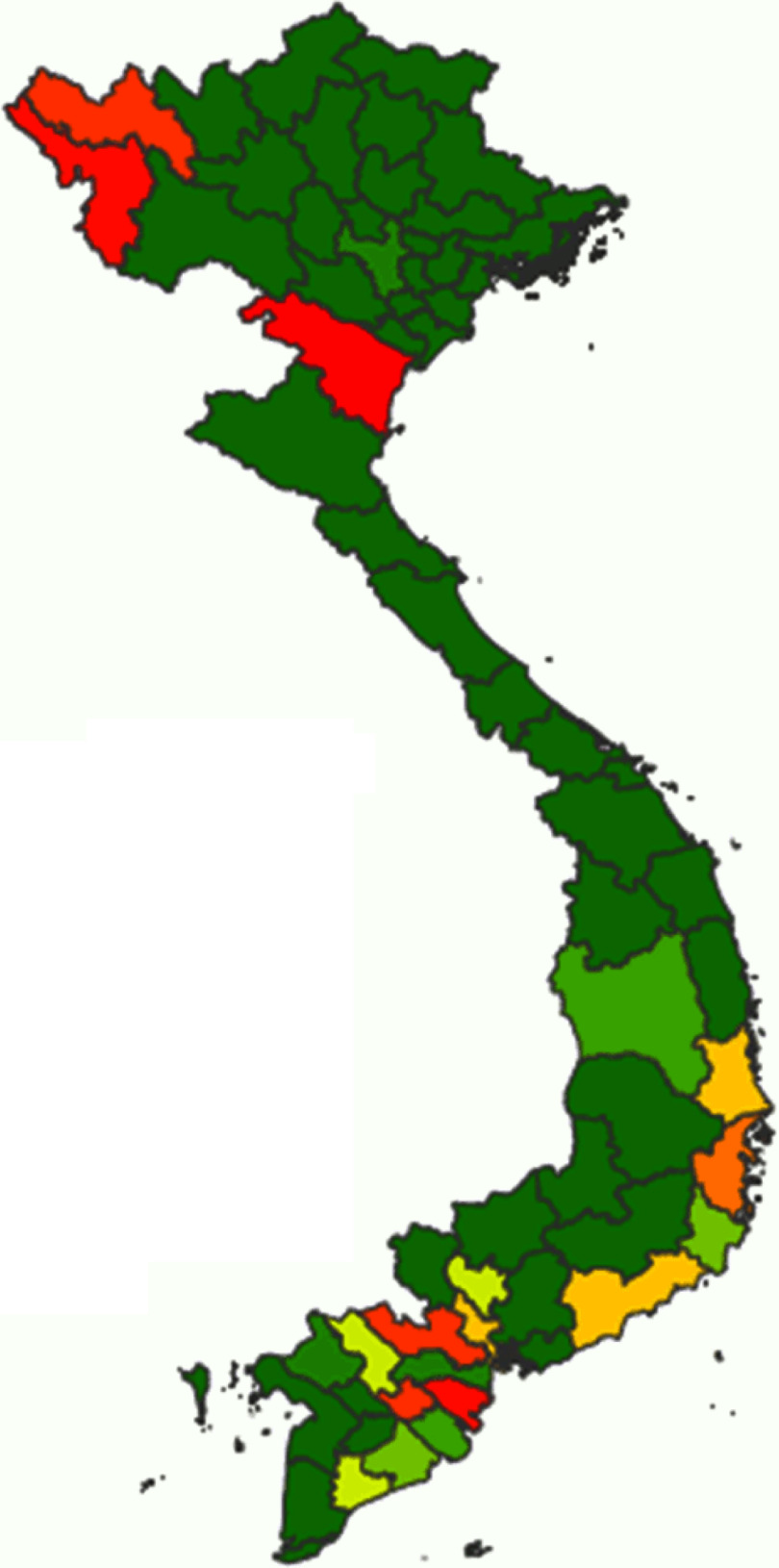
Distribution of the average consumption of fresh cassava expressed as g/capita per d in Vietnam. Cassava consumption: 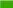, 0·00; 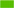, 0·00–0·06; 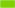, 0·06–0·21; 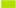, 0·21–1·14; 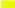, 1·14–1·63; 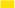, 1·63–1·70; 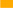, 1·70–2·55; 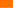, 2·55–5·66; 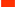, 5·66–9·65; 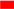, 9·65–22·09

From our calculations using the data obtained from the Vietnamese National Institute of Nutrition^(19)^, the overall median daily consumption of fresh cassava amounted to 1·14 g/capita per d (interquartile range 4·43) in the nineteen cassava-consuming provinces of Vietnam. However, there was considerable regional variation in terms of cassava consumption: data from this survey indicate that Thanh Hoa was the province with the highest reported mean consumption (22·09 g/capita per d), followed by Ben Tre, Dien Bien and Vinh Long (Fig. 2; Table 2). Interestingly, two extreme ‘clusters’ of isolated cases of very high consumption of fresh cassava (relating to twenty-one households in total) were identified (data not shown), one in each of two geographical regions (spanning nine provinces in total). One ‘cluster’, composed of six households in the ‘Northern Midlands and Mountain’ region (four households from the Dien Bien province and two from Lai Chau province), was reported to consume 336·67 g/capita per d. The other ‘cluster’, composed of fifteen households in the ‘North Central and Central Coast’ region (eight households from Thanh Hoa province, two from Quang Ngai province and one each from Phu Yen, Quang Tri, Binh Thuan, Ninh Thuan and Khanh Hoa provinces), was reported to consume 325·52 g/capita per d. The health-related implications of these isolated cases are explored in the Discussion. In the case of *dried* cassava, the reported mean daily consumption amounted to 1·27 g/capita per d in eight cassava-consuming provinces and zero in the rest of the provinces of Vietnam (data not shown).

**Table 2:** Reported the highest mean daily consumption in Vietnam, Indonesia and Philippines and calculated median values for fresh cassava *per capita*

Country	Province	Consuming areas reported	Reported mean consumption (g/capita per d)	Calculated median consumption (g/capita per d)	IQR
Vietnam^a^		19		1·14	4·43
	Thanh Hoa		22·09		
	Ben Tre		9·65		
	Dien Bien		9·10		
	Vinh Long		5·66		
	Lai Chau		4·69		
Indonesia^b^		33		9·01	8·86
	Maluku		35·79		
	Maluku Utara		31·64		
	Papua		29·47		
	Nusa Tenggara Timur		27·02		
	Sulawesi Tenggara		19·14		
Philippines^b^		83		7·28	8·43
	Tawi-tawi		72·40		
	Sulu		48·89		
	Palawan		34·15		
	Apayao		29·87		
	Surigao del Sur		28·63		

IQR, interquartile range.

*P*-values were adjusted using Bonferroni’s method.

^a,b^Statistically significant difference in fresh cassava root consumption between the three countries using Mood’s Test of Medians (*P* < 0·05), followed by *post-hoc* Mood’s Test of medians multiple comparison are indicated with superscripts.

### Reported cassava consumption in Indonesia

**Fig. 3:**
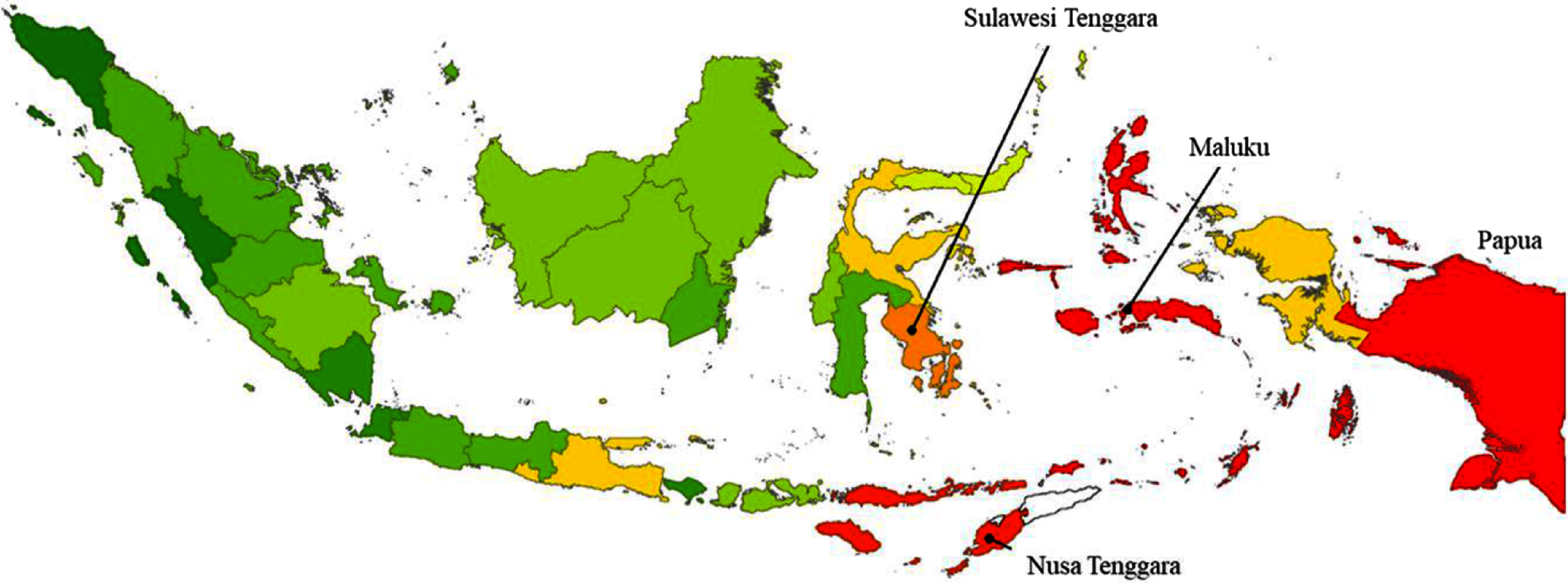
Distribution of the average consumption of fresh cassava (g/capita per d) in Indonesia. Cassava consumption: 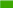, 2·44–3·47; 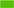, 3·47–6·62; 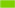, 6·62–9·62; 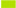, 9·62–13·59; 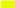, 13·59–16·28; 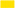, 16·28–19·14; 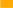, 19·14–22·17; 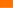, 22·17–25·13; 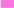, 25·13–28·13; 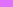, >28·13

From our calculations using the data obtained in the Indonesia National Socioeconomic Survey^(20)^, the overall median daily consumption of fresh cassava in Indonesia amounted to 9·01 g/capita per d (interquartile range 8·86), nationwide. The province with the highest reported mean daily *per capita* consumption of fresh cassava was Maluku (35·79 g/capita per d), followed by Maluku Utara, Papua, Nusa Tenggara Timur and Sulawesi Tenggara, as shown in Fig. 3. In addition, the reported mean consumption of dried cassava in twenty-nine cassava-consuming provinces in Indonesia amounted to 0·21 g*/*capita per d, with zero consumption reported in the remaining four provinces (data not shown).

### Reported cassava consumption in the Philippines

**Fig. 4:**
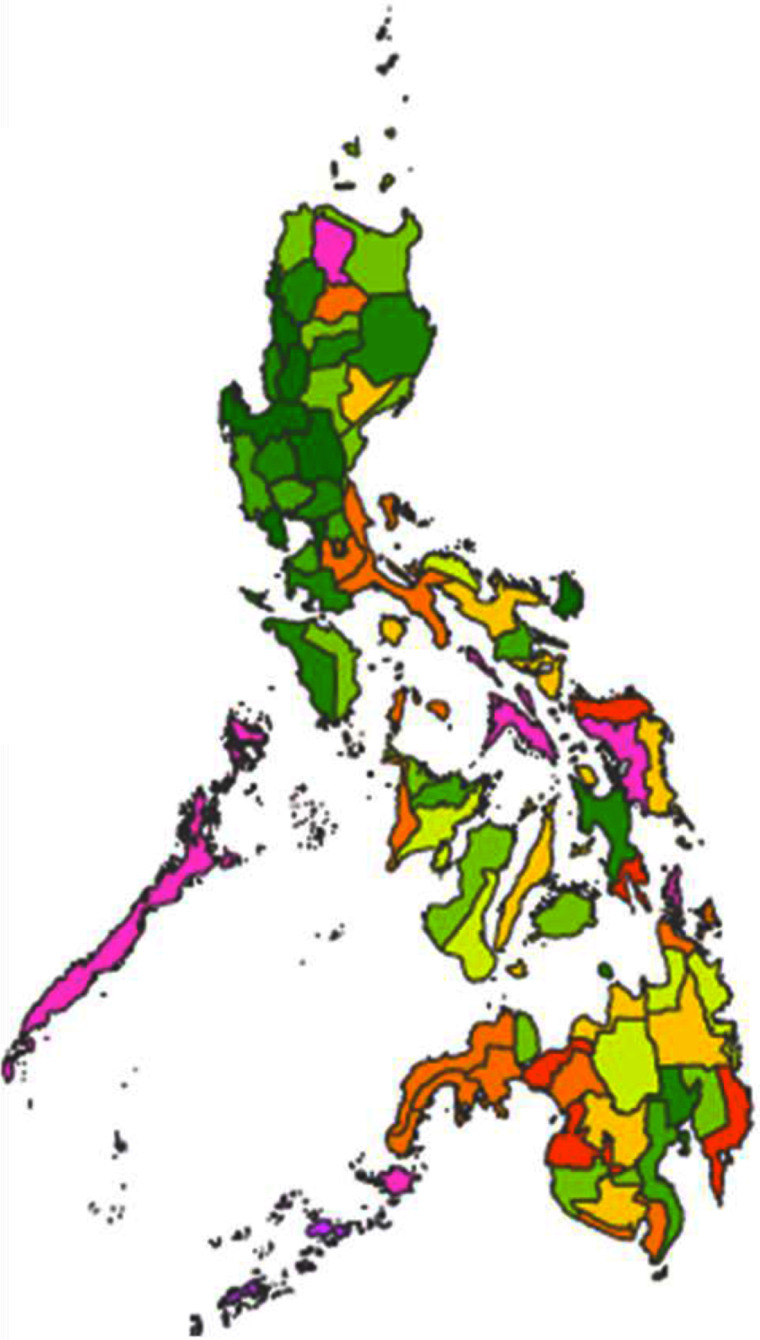
Distribution of the average *per capita* consumption of fresh cassava by provinces in the Philippines (g/capita per d). Cassava consumption: 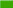, 0·00–2·68; 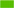, 2·81–3·79; 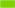, 4·19–4·97; 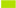, 5·36–6·71; 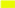, 6·87–8·04; 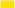, 8·55–10·42; 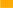, 10·42–14·92; 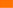, 15·37–20·95; 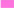, 20·95–34·15; 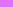, 34·15–72·40

From our calculations using data obtained from the Philippine Bureau of Agricultural Statistics^(36)^, the overall median daily consumption of fresh cassava in the Philippines amounted to 7·28 g/capita per d (interquartile range 8·43). It was found that Tawi-tawi was the province with the highest reported mean daily *per capita* consumption (72·40 g/capita per d) followed by Sulu, Palawan, Apayao and Surigao del Sur as shown in Fig. 4.

Based on the limited data available, which are reported in summary above, overall *per capita* consumption for fresh cassava in Indonesia was found to be higher than in the Philippines and in Vietnam. A Mood’s Test of Median showed that there was a statistically significant difference in fresh cassava root consumption between the three countries (*P* < 0·05), with median reported cassava intakes of 1·14 g/capita per d for nineteen cassava-consuming areas of Vietnam, 9·01 g/capita per d for Indonesia and 7·28 g/capita per d for the Philippines. Only the median consumption of fresh cassava in Vietnam was found to be significantly lower than was the case in both the Philippines (*P* < 0·05) and Indonesia (*P* < 0·05). Meanwhile, median *per capita* consumption values in the Philippines and Indonesia did not differ significantly from each other (*P* = 0·45), as shown in Table 2.

### Reported cyanogenic potential of cassava and its products in Vietnam, Indonesia and Philippines

Information in the scientific literature regarding the CNp of cassava and its products in the three South-east Asian countries featured in this paper is somewhat limited. In total, we found eight publications reporting the CNp levels of fresh, processed and/or dried cassava products: five are about Indonesian cassava, two are about cassava from the Philippines and one is about cassava from Vietnam. The findings from these sources are presented in Table 3, along with pertinent CNp limits as set out by the relevant bodies. In several instances, the reported mean CNp values violate the stated limits, including with fresh roots in Indonesia, where a mean value of 85 mg/kg HCN (fwb) was reported^(26)^, and fresh Indonesian roots on sale in Singapore which had a mean overall CNp of 59·5 mg/kg HCN (fwb)^(27)^. Similarly, in some instances the mean CNp of processed/dried Indonesian cassava products exceeded relevant limits, with mean values of 54 mg/kg HCN (fwb)^(23)^ and 56·8 mg/kg HCN (fwb) reported^(28)^.

**Table 3:** Reported cyanogenic potential (CNp; as mg/kg hydrogen cyanide, fresh weight basis) of fresh cassava roots and dried cassava products of Vietnamese, Indonesian and Philippine origin

Product	Vietnam	Indonesia		Philippines		Maximum CNp for human consumption (standard-setting authority)
			Mean		Mean	
Fresh roots (general)	No data obtained	0–60*	19	29·2–382·6†	114·7	50 mg/kg (CAC)
		0·0–268·0‡	85·0			
Fresh roots for boil-and-eat consumption	No data obtained	33·8–99·5§	59·5	No data obtained		50 mg/kg (CAC)
Frozen parenchyma for boil-and-eat consumption	Mean: 52·0║	No data obtained		11·1–33·9¶	20·2	50 mg/kg (CAC)
Dried cassava products	No data obtained	0–190*	54	No data obtained		10 mg/kg (CAC)
		30–80**	56·8			40 mg/kg (Indonesian government)
		2·4–25·2^††^	14·6			

CAC, Codex Alimentarius Commission.

* Data from twenty-seven root samples and twenty-nine samples of flour, chips and gaplek taken from four different provinces of the country, reported by Djazuli & Bradbury^(23)^.

† Data from roots of eighteen different cultivars, reported by Yeoh & Truong^(29)^.

‡ Data from roots of 179 different cultivars, reported by Hidayat *et al.*^(26)^.

§ Data from twenty-four randomly sampled roots on retail sale in Singapore, reported by O’Brien *et al.*^(27)^.

║ Data from three samples of frozen root parenchyma on retail sale in Australia, reported by Burns *et al.*^(30)^.

¶ Previously unpublished data from ten packs of frozen root parenchyma, on retail sale in Ireland, from O’Brien *et al.* study^(52)^.

** Data from four samples of cassava flour, reported by Egan *et al.*^(53)^.

†† Data from nine samples of Indonesian cassava flour and gaplek reported by O’Brien *et al.*^(28)^.

Fresh roots tested in the Philippines were reported to have had a mean CNp of 114·7 mg/kg HCN (fwb)^(29)^, while frozen cassava parenchyma of Vietnamese origin tested in Australia was reported to have a mean CNp of 52 mg/kg HCN (fwb) (Table 3)^(30)^.

## Discussion

From the (admittedly limited) data available, a significant difference was noted between the three countries’ median *per capita* consumption of fresh cassava (*P* < 0·05, Mood’s Test Medians non-parametric test). Interestingly, the consumption of fresh cassava in Vietnam was found to be significantly lower than was the case in both the Philippines and Indonesia, whose *per capita* consumption values did not differ significantly from each other. However, it is important to note that the Vietnam data were not nationally representative, as the available raw data (and thus also our results) did not represent all sixty-three provinces. In addition, when calculating national median consumption values in this review, the provincial mean values that we used have not been weighted according to provincial population; this may have biased our estimated national consumption values to some extent. Available published data regarding the CNp of cassava in the three countries are also lamentably very scarce: given the nutritional importance of cassava in certain regions/to certain human groups, this is a cause for concern.

Available information appears to indicate that cassava consumption within each country varies considerably according to region, although care is sometimes required as findings from different studies may appear to contradict each other. For example, findings published in 2001, from a small-scale study conducted in two agro-ecological settings in Vietnam, indicated that consumption of cassava among rural women in the Central Highlands was higher than that of women in the Mekong River Delta^(31)^. This appears to contradict findings, within the National Institute of Nutrition Vietnam^(19)^ data set that was used in this paper, which indicated higher *per capita* consumption of cassava in the Mekong River Delta than in the Central Highlands region (data not shown). Such discrepancies between outcomes of different studies may reflect changes in consumption patterns with time or may arise on account of differences in sampling strategy, criteria used for the recruitment and selection of study participants, and survey technique^(32)^.

Regional variations in cassava cultivation and consumption can be related to climatic factors (Nghiem in 1992 cited unfavourable weather conditions as a major inhibitory factor against the processing and consumption of cassava in northern Vietnam)^(33)^ and socio-economic factors (urban as compared with rural, as noted within the Indonesian context by Dixon in 1979 with higher consumption of cassava roots by the rural population as compared with the urban population)^(34)^. This variation in fresh cassava root consumption between urban and rural areas has also been highlighted in more recent decades in Cameroon^(35)^, Philippines^(36)^ and India^(37,38)^. Additionally, there is some published evidence which indicates that food consumption patterns may be changing in Asian countries, partly as a result of economic growth and globalised trade^(39,40)^. This may be affecting cassava consumption in some countries. For example, Abdullah^(41)^ states that in Indonesia the consumption of rice has increased, with concomitant reductions in the consumption of sago and cassava.

In the Philippines, cassava-consumption patterns may also have varied over time. From our results, we estimate that the mean energy consumption from cassava in Tawi-tawi, Sulu, Lanao del Norte, Dinagat Island and Basilan amounted to 481·16, 327·2725, 114·014, 171·3348 and 84·64232 kJ/capita per d, respectively, which represent 5·48, 3·73, 1·31, 1·95 and 0·96 %, respectively, of the total energetic intake of an adult female, considering the recommended energy intake as 8786·4 kJ per d (2008 and 2009 data, reported in 2012). In a previous iteration of the Philippines dietary survey (2007 data, reported in 2009), using the same methodology, it was reported that the province with the highest consumption of cassava was Tawi-tawi (389·0283 kJ/capita per d), followed by Lanao del Norte, Sulu, Dinagat Island and Basilan with 357·6483, 304·2605, 257·5252 and 227·9862 kJ/capita per d, respectively^(42)^. These findings might indicate an increase in the consumption of fresh cassava in Tawi-tawi, Sulu and Lanao del Norte, and decrease in Dinagat Island and Basilan, between 2007 and 2009. Alternatively, these differences may be on account of sampling error. Unfortunately, no information from previous years regarding fresh cassava consumption in Vietnam is known.

If one broadens the scope of the discussion and considers cassava consumption in other countries and continents, some interesting comparisons can be made. Still in the South of Asia, for example in some states in India, cassava is consumed as baked/cooked roots and fried chips in Kerala, Tamil Nadu, Andhra Pradesh and in the Northeastern states^(37)^. Data published in 1995 indicated that in Kerala, the average cassava consumption in rural areas was 410 g/capita per d, while that in urban areas was 120 g/capita per d^(38)^. Although this Indian study published over 20 years ago did not differentiate between fresh root consumption and the use of dried cassava products, nonetheless its findings confirmed the considerably higher cassava consumption in rural (as compared with urban) areas that was highlighted by Dixon^(34)^ in Indonesia. Taken in a global perspective, human consumption of cassava in South-east Asia appears relatively minor if compared with that of some sub-Saharan African countries, where production for human consumption has reportedly been on the increase^(43)^. In keeping with previous observations, cassava consumption in sub-Saharan Africa has been found to vary widely, and extreme weather events (e.g., drought) or socio-economic factors may sometimes contribute to ‘spikes’ in consumption^(44,45)^.

One major limitation of this study was the use of national food consumption surveys reporting the average cassava consumption based on provincial and regional means. The data from the three countries were not generated in the same way: for example, 24-h recall was used in Vietnam, while 1-week recall was used in Indonesia and the Philippines. It is noted by the authors that the comparison of cassava-consumption data based on differing recall periods requires care, as findings have indicated that longer recall period tends to be associated with higher reported consumption than is the case with a shorter recall period^(46,47)^. Consequently, it may well be that the considerably lower reported consumption of cassava in Vietnam (relative to Vietnam) is partially due to this methodological difference. In addition, the year(s) when data were obtained, population group and sample size also varied according to country. It is also worth noting that household-sourced information in all cases was collected by interviewing the head of the household about the food consumption of all household members. This could give rise to inaccuracies regarding the number of people per household consuming cassava and regarding the energy intake per person. A further limitation is the fact that information regarding the *food lists* used in the surveys was not accessible: had such information been available, it may have helped with understanding of how the information regarding cassava consumption was obtained. Nevertheless, national food consumption surveys are considered more informative than food balance sheets^(32,48)^. The most recent-published food balance sheets from FAO indicate that Indonesia is the country with the highest dietary energetic consumption from cassava, followed by the Philippines and thirdly Vietnam^(49)^. This ranking is in accordance with the findings of this paper, although the estimated cassava-consumption quantities do differ and consider the mean cassava consumption.

It is important to note that the cassava-consumption data from the three South-east Asia countries reported in this paper relate only to fresh (‘cook-and-eat’) cassava. Unfortunately, the national food consumption surveys used in this paper did not report on the consumption of various products made from cassava, such as noodles, chips or tapioca. Some of the scientific literature makes reference to the processing of cassava in Indonesia to yield fermented (‘*tape’*) or dried (‘*kripik’*) products, as well as starch and other products^(50)^. There is also mention of the inclusion of cassava in some types of cake, desserts, dry pancakes and noodles in Vietnam^(33)^. Such products are not included here, and consequently, it may be assumed that the *per capita* energetic intake derived from cassava in the three countries has been underestimated to some degree. Products made from cassava appear to have considerable market potential in ‘niche’ markets in urban centers both in Asia and further afield^(51)^.

Information regarding cassava consumption by humans in other Asian countries such as Thailand, Laos, China and India has been more difficult to obtain, partly because of a tendency for food consumption reports from those countries only to report cassava consumption as part of the overall root and tuber category, and not as an individual commodity. Other limitations encountered in official reports from these countries have included language (unavailability of information in English or other languages familiar to the authors of this paper) and limitations in the quantity or coverage of data reported in national food consumption reports. Consequently, data from India, Laos, China and Thailand have not been included in this paper. Future research could help clarify the contribution of cassava consumption to total energy intake of the two countries with the highest amount of cassava consumption reported in this paper (Indonesia and the Philippines) and the minority ethnic groups of Vietnam to ascertain the extent of a shift in consumers’ preference, including substitution of cassava with other food commodities.

Despite limitations in the data available, some of the reported cassava-consumption values do provoke concern, none more so than those relating to the small ‘extreme clusters’ of fresh cassava consumption in two geographical regions of Vietnam that have been mentioned earlier (see Results). As stated earlier, these small ‘clusters’ included households located in nine different provinces of the country regions. Among these, three of the highest-consuming provinces (Dien Bien, Lai Chau and Thanh Hoa) are included.

The rather limited published data regarding cassava CNp in the three countries yield some interesting topics for discussion. From the study conducted by Hidayat *et al.*^(26)^ on fresh roots of 179 Indonesian cultivars, the mean CNp (85 mg/kg HCN, fwb) was considerably in excess of the CAC maximum level for sweet cassava of 50 mg/kg HCN (fwb). The authors also indicated that around 60 % of the cultivars tested should not be used directly for boil-and-eat consumption, but that their roots should undergo more extensive processing, to lower their CNp instead^(26)^. In a country with a long-established culture of cassava cultivation and – indeed – processing, it appears reasonable to assume that this advice would be heeded. As regards the high mean CNp of the fresh roots of eighteen cultivars tested in the Philippines (114·7 mg/kg HCN, fwb) by Yeoh & Truong^(29)^, it is worth noting that the study itself was focused on comparing CNp-assay methods, and no commentary was provided regarding which – if any – of the cultivars were being sold to consumers, nor the intended uses of any of the roots. Thus, it is not possible to ascertain whether consumers would be exposed to those cultivars and their cyanogens. In contrast, there appears to be a clear cause for concern arising from the small random sample of Indonesian roots tested in Singapore by O’Brien *et al.*: not only did the mean overall CNp of 59·5 mg/kg HCN (fwb) exceed the aforementioned CAC maximum level, but more than half of the individual roots sampled (fifteen of twenty-four) exceeded it, with one root having a CNp of 99·5 mg/kg HCN (fwb)^(27)^. Similarly, while a random sample of ten packs of frozen parenchyma from the Philippines tested in Ireland was fully compliant with the CAC requirement for sweet cassava^(52)^, the reported CNp of 52 mg/kg HCN (fwb) in frozen Vietnamese parenchyma on retail sale in Australia tested by Burns *et al.*^(30)^ exceeds the CAC maximum level and might possibly indicate a problem.

In countries where fresh and frozen cassava roots are routinely imported, regular sampling/testing should be carried out, with the aim of ascertaining whether or not ‘bitter’ high-CNp roots are routinely on sale for boil-and-eat consumption, and whether or not they represent a significant threat to human health. As regards the data for Indonesian dried cassava products reported by Djazuli & Bradbury^(23)^ and Egan *et al.*^(53)^, not only did the reported mean CNp values clearly exceed both the CAC and Indonesian government maximum levels, but more than half of the individual samples tested also did so, and four individual samples tested by Djazuli & Bradbury exceeded 140 mg/kg HCN (fwb)^(23)^. These data are in sharp contrast with the CNp of samples of Indonesian cassava flour and gaplek tested nearly 20 years later in Singapore: at 14·6 mg/kg HCN (fwb), the mean CNp was well below the Indonesian government maximum level, and all individual samples were too^(27)^. One possible explanation for this contrast might be that processing standards (in terms of cyanogen removal) for dried cassava products may have improved in Indonesia during recent decades. Nonetheless, more sampling and CNp assay of dried cassava products should be carried out, in order to attain increased certainty as to how safe such products are.

As stated in the ‘Introduction’ section, in 2011 the joint WHO/FAO body JECFA established a PMTDI for dietary cyanide of 0·02 mg/kg body weight, with the aim of enabling the calculation of health risks^(17)^. If this is so, then a 60 kg adult may safely consume up to 1·2 mg of cyanide (or 1·25 mg of HCN) per d. Based on this, and as stated by JECFA, up to 25 g/d of fresh cassava or cassava product with a CNp of 50 mg/kg HCN (fwb) may be safely consumed by such an adult. While the assumed 60 kg body weight, as used by JECFA, is suitable for an adult population, consideration of household *per capita* cassava consumption (as is the case in the present study) implies not only adults but children and adolescents as well. In a recently published study relating to mycotoxin exposure among a maize-consuming population in South Africa, Shephard *et al.*^(54)^ used average body weights of 20 kg for children (1–9 years of age), 50 kg for adolescents (10–17 years of age) and 60 kg for adults (18–65 years of age). To represent an ‘average household person’ in the present study, it would appear reasonable to use the ‘rounded’ mean of these three body weights, that is, 43 kg body weight. Such a ‘person’ may safely consume up to 0·86 mg of cyanide (or 0·89 mg of HCN) per day and up to 17·9 g/d of fresh cassava or cassava product with a CNp of 50 mg/kg HCN (fwb). If one considers, against the backdrop of the JECFA PMTDI, some of the cassava-consumption data reported in this paper, together with some of the reported CNp values of cassava from the respective countries, then there clearly could be some risk to public health. In the Tawi-tawi province of the Philippines, with a reported mean daily consumption of fresh cassava of 72·4 g/capita per d, the cassava consumed would be required to have a CNp of 12·4 mg/kg HCN (fwb) or less, in order to comply with the PMTDI (based on an average 43 kg individual). One notes, however, from the limited CNp data assembled (see Results section) that the majority of the CNp values reported are considerably higher than this. In Table 4, some ‘scenarios’ are presented, based on reported fresh cassava-consumption values in the highest-consuming provinces of each country, and assuming a range of theoretical cassava CNp values. These comprise the CAC sweet cassava maximum level of 50 mg/kg HCN (fwb), a theoretical level of 100 mg/kg HCN (fwb) and a theoretical ‘worst-case scenario’ of 383 mg/kg HCN (fwb), which represents the highest individual CNp reported in Table 3. As shown in Table 4, the scenario involving fresh cassava with a CNp of 50 mg/kg HCN (fwb) has all of the Philippine and Indonesian provinces meeting or exceeding the PMTDI (based on a 43 kg individual), while most of the listed Vietnamese provinces are below the PMTDI. It is also worth bearing in mind that – even in those cases where a province’s mean consumption figure is consistent with a value below the PMTDI – nonetheless some of the households within that province would be consuming more than the provincial mean, and thus could possibly be meeting or exceeding the PMTDI. The ‘100 mg/kg’ scenario has the majority of the featured provinces exposed to more than thrice (and up to eight times) the PMTDI. With the ‘383 mg/kg’ worst-case scenario, most provinces would have over ten times (and up to over thirty times) the PMTDI. Most worrying of all would be the small-scale ‘extreme clusters’ reported in Vietnam (six households in ‘Northern Midlands and Mountain’ region and fifteen households in ‘North Central and Central Coast’ region), where, as shown in Table 4, the ‘50 mg/kg’ scenario corresponds to over eighteen times the PMTDI, while the ‘100 mg/kg’ scenario corresponds to over thirty-six times, and the ‘383 mg/kg’ worst-case scenario corresponds to over 140 times the PMTDI.

**Table 4:** Theoretical hydrogen cyanide consumption *per* person (mg) according to cassava cyanogenic potential (CNp) and the Provisional Maximum Tolerable Daily Intake (PMTDI) in the provinces or regions that reported the highest levels of cassava consumption

Country/province/region	Reported mean consumption (g/capita per d)	Theoretical daily *per capita* HCN consumption (mg) according to the respective stated cassava CNp, and corresponding multiple of PMTDI ingested that this represents					
		HCN intake (mg) if CNp = 50 mg/kg HCN (fwb)	How many PMTDI this represents	HCN intake (mg) if CNp = 100 mg/kg HCN (fwb)	How many PMTDI this represents	HCN intake (mg) if CNp = 383 mg/kg HCN (fwb)	How many PMTDI this represents
Vietnam							
Extreme cluster 1 N. Midlands and Mountain (six households)	336·7	16·8	18·9	33·7	37·8	128·9	144·9
Extreme cluster 2 N. Central and Central Coast (fifteen households)	325·5	16·3	18·3	32·5	36·6	124·7	140·1
Thanh Hoa	22·1	1·1	1·2	2·2	2·5	8·5	9·5
Ben Tre	9·7	0·5	0·5	1	1·1	3·7	4·2
Dien Bien	9·1	0·5	0·5	0·9	1·0	3·5	3·9
Vinh Long	5·7	0·3	0·3	0·6	0·6	2·2	2·4
Indonesia							
Maluku	35·8	1·8	2·0	3·6	4·0	13·7	15·4
Maluku Utara	31·6	1·6	1·8	3·2	3·6	12·1	13·6
Papua	29·5	1·5	1·7	2·9	3·3	11·3	12·7
Nusa Tenggara Timur	27·0	1·4	1·5	2·7	3·0	10·3	11·6
Sulawesi Tenggara	19·1	1·0	1·1	1·9	2·2	7·3	8·2
Philippines							
Tawi-tawi	72·4	3·6	4·1	7·2	8·1	27·7	31·2
Sulu	48·9	2·4	2·7	4·9	5·5	18·7	21·0
Palawan	34·2	1·7	1·9	3·4	3·8	13·1	14·7
Apayao	29·9	1·5	1·7	3·0	3·4	11·4	12·9
Surigao del Sur	28·6	1·4	1·6	2·9	3·2	11·0	12·3

HCN, hydrogen cyanide.

The PMTDI of 0.89 mg of HCN was derived based on an estimated household-representative body weight of 43 kg, this being the rounded arithmetical mean of the average body weights of an adult, an adolescent and a child.

The CNp level of 50 mg/kg (fwb) is the maximum level for sweet cassava as defined by the Codex Alimentarius Commission^(24)^; the 100 mg/kg (fwb) level is considered ‘realistic’ based on the data reported in Table 3; the 383 mg/kg (fwb) level is the ‘worst-case scenario’, again based on the data reported in Table 3.

While the depicted ‘worst-case scenario’ seems rather unlikely (its theoretical cyanide exposure has been calculated using the CNp of roots obtained at a cassava breeding/experimental station)^(26,29,55)^, the scenario based on roots with CNp 100 mg/kg certainly appears credible in view of the data reported in Table 3, and the implication would be that populations in several provinces of the Philippines and Indonesia, and one province of Vietnam, are consuming upward of around three times the PMTDI of cyanide (not to mention the two small ‘extreme clusters’ in Vietnam, where over thirty-six times the PMTDI may be consumed). There does appear to be an urgent need to implement regular sampling and testing of the CNp of ‘market-ready’ cassava and cassava products in each of the three countries.

One final aspect to mention is that of dietary protein intake. As stated earlier in this paper, cassava-consuming groups with an inadequate protein intake may be vulnerable to particular problems, such as growth retardation, owing to amino-acid depletion through the body’s conversion of cyanide into thiocyanate. In Indonesia, Maluku Utara province is reported to have a high prevalence of wasted and stunted children^(56)^, together with a mean reported cassava consumption of 31·64 g/capita per d: depending on the CNp of the cassava consumed, it is not inconceivable that cassava may be contributing to these problems in the case of some children with an already poor dietary protein intake. It should be noted that even when a provincial mean is not indicative of an exposure to cyanide above the Codex norm, some households within the province could still have consumption levels above the norm.

## Conclusion

In conclusion, the (admittedly limited) data available indicate that the populations of the Philippines and Indonesia consume more cassava *per capita* than the population of Vietnam. Nonetheless, the data reported from each country are not as consistent and as clear as one would like. From a dietary perspective, it would be helpful if the data collected were to include information regarding food processing for each commodity, and the complete food list used in each food consumption survey should be reported. We also recommend the reporting not only of mean consumption values for each commodity but also the accompanying sd, to facilitate a clearer idea as to the nature of the data regarding consumption. While it appears that cassava consumption in Vietnam and the Philippines may be increasing, it appears to be decreasing in Indonesia.

From a food safety perspective, if the cassava utilisation continues to decrease the food safety issue might be solved by itself. However, the data regarding the CNp of cassava and its products being consumed in the three countries are limited; the evidence suggests that the inhabitants of several provinces of these countries are ingesting 3–8 times the PMTDI of cyanide. There appears to be a pressing need for both accurate food intake surveys (to facilitate measurement of the intake of cassava and its products) and compositional studies (to provide accurate data regarding the CNp of the cassava being consumed), thus enabling the accurate assessment of dietary exposure to cyanide. If limited resources do not allow for large-scale surveys, the focus should be on ‘priority groups’ such as infants and expectant mothers. As a general point, there is evidently a need for more extensive CNp testing of cassava roots and products that are destined for human consumption.

## References

[1] Food and Agriculture Organization, World Food Programme & International Fund for Agricultural Development (2018) The state of food security and nutrition in the world 2018 – UNICEF DATA. https://data.unicef.org/resources/sofi-2018/ (accessed November 2018).

[2] Langendorf C, Roederer T, de Pee S et al. (2014) Preventing acute malnutrition among young children in crises: a prospective intervention study in Niger. PLoS Med 11, e1001714. 25180584 10.1371/journal.pmed.1001714 PMC4152259

[3] Wakeel A, Farooq M, Bashir K et al. (2018) Micronutrient malnutrition and biofortification: recent advances and future perspectives. In Plant Micronutrient Use Efficiency, pp. 225–244 [A Wakeel, M Farooq, K Bashir et al., editors]. London: Elsevier.

[4] Warr P (2013) Food Security, Agriculture, and Poverty in Asia. http://citeseerx.ist.psu.edu/viewdoc/download;jsessionid=27742DB3598B4381E0A423ED227A53D2?doi=10.1.1.434.8906&rep=rep1&type=pdf (accessed November 2019).

[5] Howeler R, Thomas N, Holst Sanjuán K et al. (2013) Save and Grow: Cassava. A Guide to Sustainable Production Intensification, Produire Plus Avec Moins, Ahorrar Para Crecer. Roma, Italy: FAO.

[6] United States Department of Agriculture (USDA). Basic Report 11134, Cassava, Raw. https://ndb.nal.usda.gov/ndb/foods/show/2907?manu=%26fgcd=%26ds= (accessed November 2018).

[7] Barratt N, Chitundu D, Dover O et al. (2006) Cassava as drought insurance: food security implications of cassava trials in Central Zambia. Agrekon 45, 106–123.

[8] Food Agriculture Organization of United Nations Statistics (FAOSTATS) (2016) Commodity balances. http://www.fao.org/faostat/en/#data/QC/visualize (accessed December 2017).

[9] Riis L, Bellotti AC, Bonierbale M et al. (2003) Cyanogenic potential in cassava and its influence on a generalist insect herbivore Cyrtomenus bergi (Hemiptera: cydnidae). J Econ Entomol 96, 1905–1914. 14977132 10.1093/jee/96.6.1905

[10] Bennet RN & Wallsgrove RM (1994) Secondary metabolites in plant defence mechanisms. New Phytol 127, 617–633. 33874382 10.1111/j.1469-8137.1994.tb02968.x

[11] Cliff J, Muquingue H, Nhassico D et al. (2011) Konzo and continuing cyanide intoxication from cassava in Mozambique. Food Chem Toxicol 49, 631–635. 20654676 10.1016/j.fct.2010.06.056

[12] Codex Alimentarius Commission (2019) Joint FAO/WHO Food Standards Programme. Codex Committee on Contaminants in Foods, 13th Session. Discussion paper on the establishment of MLs for HCN in cassava and cassava based products and the occurrence of mycotoxins in these products, pp. 1–26. www.fao.org/fao-who-codexalimentarius/sh-proxy/en/?lnk=1&url=https%253A%252F%252Fworkspace.fao.org%252Fsites%252Fcodex%252FMeetings%252FCX-735–13%252FWDs%252Fcf13_14e.pdf (accessed July 2019).

[13] McMahon JM, White WLB & Sayre RT (1995) Cyanogenesis in cassava (*Manihot esculenta* Crantz). J Exp Bot 46, 731–741.

[14] Mlingi N, Poulter N & Rosling H (1992) An outbreak of acute intoxications from consumption of insufficiently processed cassava in Tanzania. Nutr Res 12, 677–687.

[15] Banea-Mayambu JP, Tylleskär T, Tylleskär K et al. (2000) Dietary cyanide from insufficiently processed cassava and growth retardation in children in the Democratic Republic of Congo (formerly Zaire). Ann Trop Paediatr 20, 34–40. 10824211 10.1080/02724930092048

[16] Codex Alimentarius Commission (2009) Joint FAO/WHO Food Standards Programme. Codex Committee on Contaminants in Foods, 3rd Session. Discussion paper on cyanogenic glycosides. 9, 23.

[17] World Health Organization (2011) *Evaluation of Certain Food Additives and Contaminants: Seventy-fourth (74th) Report of the Joint FAO/WHO Expert Committee on Food Additives (JECFA). WHO Technical Report Series* no. 966. Geneva: WHO. https://apps.who.int/iris/handle/10665/44788 (accessed November 2018).

[18] Brimer L (2010) Cyanogenic glycosides in food, feeding stuffs and green medicine. In Bioactive Compounds in Plants Benefits and Risks for Man and Animals, pp. 125–143 [A Bernhoft, editor]. Proceedings from a symposium held at the Norwegian Academy of Science and Letters, Oslo, 13–14 November 2008.

[19] Vietnamese National Institute of Nutrition (2010) General Nutrition Survey 2009–2010. Hanoi, Vietnam: Medical Publishing House.

[20] National Socioeconomic Survey (2014) Consumption of Calorie and Protein of Indonesia and Province. Jakarta, Indonesia: Badan Pusat Statistik.

[21] Bureau of Agricultural Statistics (2012) Annual *Per Capita* Consumption of Agricultural Commodities, Provinces, Reference Period and Classification of Baragays Statistics. http://countrystat.psa.gov.ph/selection.asp (accessed November 2018).

[22] World Health Organization (WHO) (1993) Cyanogenic glycosides. http://www.inchem.org/documents/jecfa/jecmono/v30je18.htm (accessed November 2018).

[23] Djazuli M & Bradbury JH (1999) Cyanogen content of cassava roots and flour in Indonesia. Food Chem 65, 523–525.

[24] Food and Agriculture Organization/World Health Organization (2013) Codex standard for sweet cassava. Food and Agriculture Organization and World Health Organization of the United Nations, Rome, Italy 238–2003, 1–4.

[25] Ayetigbo O, Latif S, Abass A et al. (2018) Comparing characteristics of root, flour and starch of biofortified yellow-flesh and white-flesh cassava variants, and sustainability considerations: a review. Sustainability 10, 1–32.

[26] Hidayat A, Zuaraida N, Hanarida I et al. (2000) Cyanogenic content of cassava root of 179 cultivars grown in Indonesia. J Food Compos Anal 13, 71–82.

[27] O’Brien GM, Ong YL, Koh RAN et al. (2018) Snapshot Survey of the Cyanogenic Potential of Fresh Cassava Roots on Retail Sale in Singapore. Cassava Transformation in Africa. IVth International Cassava Conference – GCP21-IV, Cotonou, Benin. http://www.gcp21.org/beninconference/ (accessed November 2018).

[28] O’Brien GM, Toh KH, Lim BJ et al. (2018) Snapshot Survey of the Cyanogenic Potential of Cassava Products on Retail Sale in Singapore. Cassava Transformation in Africa. IVth International Cassava Conference – GCP21-IV, Cotonou, Benin. http://www.gcp21.org/beninconference/video.html (accessed November 2018).

[29] Yeoh HH & Truong VD (1993) Quantitative analysis of Linamarin in cassava using a cassava β-glucosidase electrode. Food Chem 47, 295–298.

[30] Burns AE, Bradbury JH, Cavagnaro TR et al. (2012) Total cyanide content of cassava food products in Australia. J Food Compos Anal 25, 79–82.

[31] Ogle BM, Hung PH & Tuyet HT (2001) Significance of wild vegetables in micronutrient intakes of women in Vietnam: an analysis of food variety. Asia Pacific J Clin Nutr 10, 21–30. 10.1046/j.1440-6047.2001.00206.x 11708605

[32] Serra-Majem L, MacLean D, Ribas L et al. (2003) Comparative analysis of nutrition data from national, household, and individual levels: results from a WHO-CINDI collaborative project in Canada, Finland, Poland, and Spain. J Epidemiol Commun Health 57, 74–80. 10.1136/jech.57.1.74 PMC1732273 12490653

[33] Atthasampunna P (1990) Cassava processing and utilization in Thailand. In *Regional Workshop on Cassava Breeding, Agronomy and Utilization Research in Asia (3, 1990, Malang, Indonesia)* [RH Howeler, editor]. Cassava Breeding, Agronomy and Utilization Research in Asia: Proceedings.

[34] Dixon J (1979) Production and consumption of cassava in Indonesia. Bull Indones Econ Stud 15, 83–106.

[35] Mennen LI, Mbanya JC, Cade J et al. (2000) The habitual diet in rural and urban Cameroon. Eur J Clin Nutr 54, 150–154. 10694786 10.1038/sj.ejcn.1600909

[36] Bureau of Agricultural Statistics (2010) Survey of Food Demand for Agricultural Commodities in the Philippines. https://psa.gov.ph/sites/default/files/SFD_Volume1_May2010.pdf (accessed November 2019).

[37] Edison S & Srinivas T (2006) Status of cassava in India an overall view. *Crops*. Central Tuber Crops Research Institute, Indian Council of Agricultural Research.

[38] Nayar TV, Kabeerathumma S, Potty V et al. (1993) Recent progress in cassava agronomy research in India. In *Cassava Breeding, Agronomy Research and Technology Transfer in Asia: Proceedings of the Fourth Regional Workshop held in Trivandrum, Kerala, India*.

[39] Pingali P (2007) Westernization of Asian diets and the transformation of food systems: implications for research and policy. Food Policy 32, 281–298.

[40] Popkin BM (2001) Nutrition in transition: the changing global nutrition challenge. Asia Pac J Clin Nutr 10, S13–S18. 11708577

[41] Abdullah S (2014) Local community resilience in the context of global climate change: a case from Maluku Indonesia. Sociol Anthropol 2, 309–316.

[42] Bureau of Agricultural Statistics (2008) Annual *per capita* consumption of agricultural commodities, provinces, reference period and classification of Baragays. *Statistics*. http://countrystat.psa.gov.ph/selection.asp (accessed November 2018).

[43] Nhassico D, Muquingue H, Cliff J et al. (2008) Rising African cassava production, diseases due to high cyanide intake and control measures. J Sci Food Agric 88, 2043–2049.

[44] Armah FA, Odoi JO, Yengoh GT et al. (2011) Food security and climate change in drought-sensitive savanna zones of Ghana. Mitig Adapt Strateg Glob Chang 16, 291–306.

[45] Sagoe R (2006) Climate change and root crop production in Ghana. A report prepared for Environmental Protection Agency (EPA), Accra-Ghana.

[46] Thompson FE & Byers T (1994) Dietary assessment resource manual. J Nutr 124, 2245s–2317s. 7965210 10.1093/jn/124.suppl_11.2245s

[47] Kowalkowska J, Slowinska MA, Slowinski D et al. (2013) Comparison of a full food-frequency questionnaire with the three-day unweighted food records in young Polish adult women: implications for dietary assessment. Nutrients 5, 2747–2776. 23877089 10.3390/nu5072747 PMC3738998

[48] Naska A, Berg MA, Cuadrado C et al. (2009) Food balance sheet and household budget survey dietary data and mortality patterns in Europe. Br J Nutr 102, 166–171. 18986595 10.1017/S000711450809466X

[49] Food and Agriculture Organization (2013) FAOSTAT Statistical Database 2017. http://www.fao.org/faostat/en/#data/FBS (accessed November 2018).

[50] Balagopalan C (2002) Cassava utilization in food, feed and industry. In Cassava Biology, Production and Utilization, pp. 301–318 [RJ Hillocks, JG Thresh & AC Bellotti, editors]. Oxon, UK: CABI Publishing.

[51] Howeler R (2006) Cassava in Asia: trends in cassava production, processing and marketing. Partnership in Modern Science to Develop a Strong Cassava Commercial Sector in Africa and Appropriate Varieties by 2020, pp. 1–38.

[52] O’Brien GM, Weir RR, Moody K et al. (2013) Cyanogenic potential of fresh and frozen cassava on retail sale in three Irish cities: a snapshot survey. Int J Food Sci Technol 48, 1815–1821.

[53] Egan SV, Yeoh HH & Bradbury JH (1998) Simple picrate paper kit for determination of the cyanogenic potential of cassava flour. J Sci Food Agric 76, 39–48. 10.3109/09637489809089388 9713579

[54] Shephard GS, Burger HM, Rheeder JP et al. (2019) The effectiveness of regulatory maximum levels for fumonisin mycotoxins in commercial and subsistence maize crops in South Africa. Food Control 97, 77–80.

[55] Whankaew S, Poopear S, Kanjanawattanawong S et al. (2011) A genome scan for quantitative trait loci affecting cyanogenic potential of cassava root in an outbred population. BMC Genomics 12, 266–277. 21609492 10.1186/1471-2164-12-266 PMC3123654

[56] United Nations International Children’s Emergency Fund/World Health Organization/World bank (2017) Joint Child Malnutrition Estimates – Levels and Trends (2015 edition). World Health Organization.

